# Effects of Snail Density on Growth, Reproduction and Survival of *Biomphalaria alexandrina* Exposed to *Schistosoma mansoni*

**DOI:** 10.1155/2010/186792

**Published:** 2010-06-08

**Authors:** T. D. Mangal, S. Paterson, A. Fenton

**Affiliations:** Department of Biological Sciences, University of Liverpool, Biosciences Building, Crown Street, Liverpool L69 7ZB, UK

## Abstract

The effects of snail density on *Biomphalaria alexandrina* parasitized with *Schistosoma mansoni* were investigated. Laboratory experiments were used to quantify the impact of high density on snail growth, fecundity, and survival. Density-dependent birth rates of snails were determined to inform mathematical models, which, until now, have assumed a linear relationship between density and fecundity. The experiments show that the rate of egg-laying followed a negative exponential distribution with increasing density and this was significantly affected by exposure to parasitic infection. High density also affected the weight of snails and survival to a greater degree than exposure to parasitic infection. Although snail growth rates were initially constrained by high density, they retained the potential for growth suggesting a reversible density-dependent mechanism. These experimental data can be used to parameterise models and confirm that snail populations are regulated by nonlinear density-dependent mechanisms.

## 1. Introduction

The density of conspecific individuals in the environment is a critical ecological factor that can affect the growth, survivorship, and fecundity of individuals and the consequent dynamics of populations [1–3]. Such factors become important for controlling host-parasite systems where the presence and magnitude of population regulatory factors are crucial for disease dynamics and the design of effective long-term control strategies [4]. In particular, control efforts may be negated if there is strong density dependence acting within the host-parasite system [4]. Schistosomiasis is a parasitic disease caused by digenetic trematodes, belonging to the Schistosomatidae family. It is currently endemic in 74 developing countries, infecting an estimated 200 million people [5, 6]. Schistosomiasis caused by *Schistosoma mansoni* is a major public health problem and repeated exposure can lead to liver damage and anaemia, particularly in children [6]. It is primarily found in rural areas in tropical and subtropical countries and infects humans and other vertebrates, using freshwater snails of the genera *Biomphalaria *as intermediate hosts. Transmission relies upon natural water polluted with human excreta, often found in areas of poverty or low income, where a lack of facilities forces people to use natural water bodies for domestic, recreational, occupational, or religious purposes. One proposed method of controlling schistosomiasis is to treat water bodies with a molluscicide to reduce the number of snail intermediate hosts, thereby breaking the disease transmission cycle [7, 8]. However, such control efforts may prove less effective if the snail population is regulated by density-dependent constraints that buffer it against the additional imposed mortality. 

Both laboratory and in situ studies have shown that snail density can greatly affect population growth [1, 9, 10] and this is likely to arise through resource competition (either through exploitation or possibly interference competition) rather than the increase of water-borne excretory/secretory products at high densities [2]. Previous studies have shown an inhibition of respiratory capacity at high densities, a lower capacity to consume ^59^Fe, and adverse effects on growth and fecundity [11–13]. However, it is likely that the competitive ability of snails, and their response to crowding, will also be influenced by parasitism itself, which has well-documented effects on individual snail fitness [14–17]. The interaction between parasitism and density is likely to have important implications for snail population size, schistosome epidemiology, and response of snail populations to imposed control measures [4, 18, 19]. To date, however, few studies have quantified this interaction.

Here the density-fitness relationships for the schistosome intermediate host *Biomphalaria alexandrina* are quantified, for both naïve (unexposed) snails and snails exposed to miracidia of *Schistosoma mansoni*. Data on survival, fecundity, and weight were recorded and used to determine whether density dependence affects fitness of snails.

## 2. Methods

### 2.1. Study System

*Biomphalaria alexandrina* is a freshwater snail that is an intermediate host for *Schistosoma mansoni* in shallow parts of the Nile, Egypt. The strains used in this study were from Egypt, and maintained in laboratories for approximately 20 generations. Stock colonies of *B. alexandrina* were maintained in the laboratory for approximately 20 generations without parasite pressure. The parasite was a mixed strain *Schistosoma mansoni* line (supplied by Professor Mike Doenhoff, University of Bangor/University of Nottingham), developed over 10 generations using isolates from Brazil, Egypt and Puerto Rico and routinely passaged through *Biomphalaria alexandrina* and CBA/CA mice. This combination of host and parasite ensured a high degree of compatibility (>85%) whilst minimising potential heterosis effects, which may occur with novel parasite-host combinations [20]. All snails were kept in plastic, 5 L tanks in a 12L : 12D photoperiod room (using 6 W fluorescent bulbs) at 23–25°C with <10 snails L^−1^ water. The snails were fed high-protein rabbit food pellets daily (Pascoe's Bunny Balance, Driffield, UK), and water was supplemented with 1 g calcium carbonate once every two weeks (see the study by Lewis et al. in [21] for cultivation methods).

### 2.2. Experimental Design

One hundred and sixty juvenile snails (4–7 mm) were randomly assigned to 8 experimental tanks containing water at 25°C. The tanks were equally divided into four density treatments, 5, 15, 25, and 35 snails in 2 L water, and each treatment was replicated twice. An additional 80 juvenile snails were individually exposed to 5 freshly hatched *S. mansoni* miracidia in 2 mL water for 2 h and assigned to one of the four treatments. These snails were monitored weekly for development of infection. 

All snails were transferred to incubators with a fluorescent low-light system (6 W) set to 12L : 12D. Water temperature stayed at 25°C ± 1°C throughout the experiment and was changed weekly. Before the experiment, all snails had received rabbit food *ad libitum*, and any excesses of food were removed daily. They were all, therefore, in similar states of health before introduction to the experimental tanks. During the experiment, each tank received 4 g rabbit food at 6 am, daily. Fecundity, measured as the number of eggs produced per snail per week, was recorded weekly, and eggs were removed from the tanks after counting. This was to control for any possible effects associated with a reduced surface area available for the snails to lay eggs. Note that we present fecundity data as the average numbers of eggs laid per snail over the study period. Data on weekly egg production are not presented here for simplicity. Growth, measured as wet weight, and weekly mortality rates were also recorded throughout the study. All dead snails were removed from tanks. After ten weeks, all snails were removed and weighed, and final mortality rates were recorded.

### 2.3. Statistical Analysis

Survival rates were modelled using two-way ANOVA using the “aov” function in *R* (http://www.r-project.org) to detect any significant differences between treatment groups. Linear mixed effects (LMEs) models were used to analyse the effects of exposure to miracidia and density on the weight and fecundity of snails and fitted by maximum likelihood using the lme4 package in *R*. LMEs models were used to control for pseudoreplication by adding experimental tank as a random effect. The significance of individual terms was assessed by deletion testing, that is, the difference in 2 × log  likelihood of nested models compared against a *χ* ^2^ distribution [22].

## 3. Results

### 3.1. Survivorship

**Figure 1:**
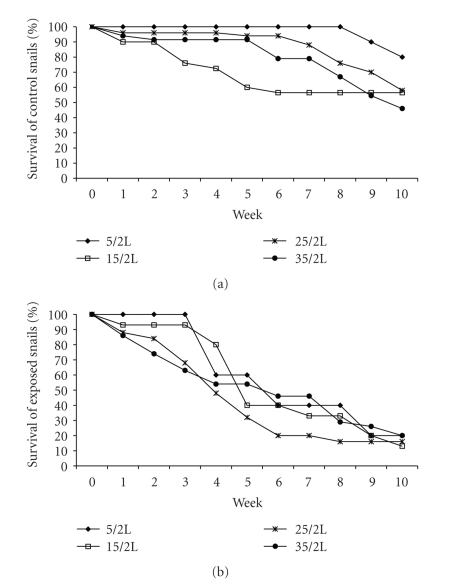
Mean percentage survival of unexposed snails (a) and snails exposed to miracidia (b). Values refer to the mean combined survival rates of snails in each treatment group. There was no significant difference between the replicates of each treatment group.

The survival rates for the unexposed snails were higher in the low-density treatments; no snails in the lowest-density group died until day 60, whereas the other three treatment groups had lower survival rates from week 1 (Figure 1). The snails exposed to *S. mansoni* miracidia showed lower survival rates than the control snails: 17% of exposed snails survived the ten-week study period whereas 60% of control snails survived. Survival was significantly affected by exposure to infection (two-way ANOVA; *P* < .0001). In contrast, the differences between density groups were not statistically significant (two-way ANOVA; *P* = .072 for density group). There were significant interactions between exposure to parasites and the density of snails (*P* < .05) suggesting that the survival of snails exposed to miracidia responded to population density in a different manner from that of unexposed snails, although there is no consistent effect of density level.

### 3.2. Weight of Snails

**Figure 2:**
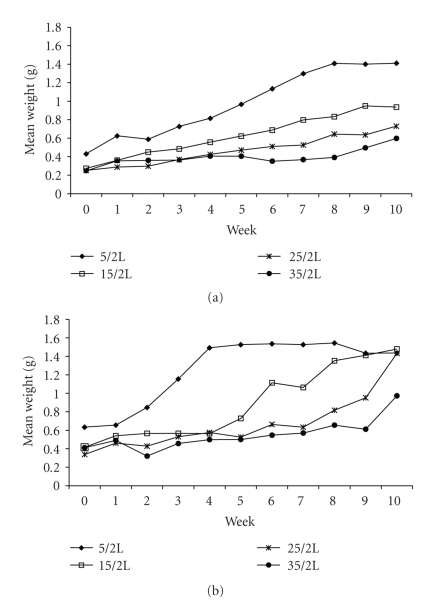
Mean weight per unexposed (a) and exposed snails (b) over ten weeks. Values for mean weight per snail refer to the average weights of all snails in each treatment group. No significant variation between tanks in each treatment group was observed.

The initial mean weights of both the unexposed and exposed snails were not significantly different (Figure 2). Thereafter, the relative growth rate slowed in both treatment groups with increasing density of snails in each tank. The minimal adequate model developed by deletion testing using linear mixed effects models is shown in Table 1. The growth of snails was negatively affected by density and this effect became increasingly severe over time. Snails exposed to parasitism in the lowest density group showed an initial rise in mean weight relative to the unexposed groups, although this effect tapered off after week 5 and was nonsignificant over the study period.

### 3.3. Fecundity of Snails

**Figure 3:**
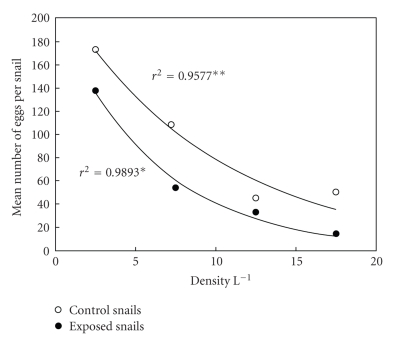
Regression analyses of the mean number of eggs produced by each snail over the 10-week study period. Values were averaged for all snails in each treatment group. Regression analysis was performed using an exponential decay model. *Exposed treatment, mean number of eggs per snail = 208.7 × exp(−0.0797 × Density). **Unexposed treatment, mean number of eggs per snail = 231.03 × exp(−0.0524 × Density).

In both the exposed and unexposed treatments there was a reduction in the number of eggs laid per snail with increasing density (Figure 3). The best-fit trendline describing these data (assessed using *R* ^2^ values) was nonlinear, following a negative exponential relationship. Snails in the unexposed treatment at the lowest density produced on average around 4 times as many eggs per week as those in the highest density. The rates of egg-laying for the snails exposed to infection followed the same pattern throughout the study period, although the unexposed snails consistently laid higher numbers of eggs (20%–69% higher). Time, density, and exposure to parasitic infection reduced the number of eggs produced by snails (Table 2).

## 4. Discussion

Exposing juvenile snails to *S. mansoni* miracidia had a negative impact on *Biomphalaria alexandrina* survival, reducing the survival rates in every group by 20%–60%. Although the miracidia did not mature within the snail hosts, the impact of exposure alone on host survival was significant. The lowest-density group showed the highest rate of growth and produced the highest egg numbers per week, contradicting other studies where the highest-density groups showed the highest rates of growth and reproduction [1]. 

*B. alexandrina* exposed to parasites showed consistently lower egg production at every density and every time-point compared with control snails, conflicting with a study by Thornhill et al., which demonstrated increased fecundity and growth in parasitized *Biomphalaria glabrata* 2 weeks post infection [14]. The difference between our results and those of Thornhill et al. is not clear, and may simply be due to variability between the species and strains used; in studies using other species combinations, infection with *S. mansoni* has been shown to lead to reduced fecundity in *Biomphalaria pfeifferi* by day 10 and a cessation in reproductive activity by day 14 post exposure [23]. Where a reduction in fecundity in parasitized snails is observed (as in our study), this has been explained by a partial or complete castration effect indirectly caused by developing parasites which sequesters hosts' resources [24, 25].

 The snails exposed to miracidia had higher mean weights compared with the control snails over the study period although this effect was not significant. This is comparable to findings by Thornhill et al., who saw a significant increase in mean diameter of exposed B. *glabrata* 28 days post exposure [14]. Exposed snails were the same average weight in each of the treatment groups at the end of the study, excluding the highest-density group. One possibility is that towards the end of the experiment the surviving snails in the “exposed” group had not actually been infected, and so were able to achieve growth rates the same as the unexposed snails. However, an alternative possibility is that the impact of parasitism on snail growth may have been mediated by the effects of higher densities, and as the higher mortality rates within the exposed groups reduced the density of snails throughout the experiment, the average weight of snails in the higher-density groups increased. Therefore, although snail growth may initially have been inhibited by high density, the snails retained the potential for growth and, as densities reduced over time, were able to compensate for deficiencies early in life to catch up with their maximal attainable size as observed in the lowest-density treatments. 

This study focused on quantifying the effects of density dependence and so the biological explanations for these effects were beyond the scope of this study. However, there are three plausible explanations for the negative feedback effects seen in the highest-density groups: competition for food resources, depletion of oxygen and/or calcium, and the production of growth inhibitory or toxic factors. The same amount of food was added to each tank daily to observe whether food would be a limiting factor and how this competition would affect survival, growth, and fecundity. The tanks were continually aerated to provide sufficient oxygen for snail growth and calcium carbonate was added weekly. *Biomphalaria glabrata* removes calcium from the water at a rate proportional to the absolute growth of the snails; it can, therefore, become a limiting factor if the concentration drops below 40–80 *μ*g mL^−1^ [26]. However, 80% of the calcium required by the snails is taken directly from the water [1]. Under these circumstances, it may be possible that calcium was limited in the higher-density tanks and growth was restricted. The third factor that may affect growth is the production of various factors by the snails themselves. Chemicals released by snails may be concentrated in high-density tanks and have previously been shown to inhibit respiration [11]. As these inhibitory factors in the tanks become more concentrated, the limiting effect would increase. Coelho et al. noted a difference in the amount of ^59^Fe uptake by snails in crowded tanks, which is necessary for snail growth [12]. It is possible, therefore, that inhibition of snail growth was caused both by the chemicals released by the snails as waste products and by a reduced capacity to uptake essential minerals, although the water in each tank was replaced weekly to minimise any adverse effects of waste product buildup. 

The density-dependent reduction of snail fecundity observed in both the exposed and unexposed groups of snails followed a nonlinear, negative exponential relationship. This pattern differs from the traditional linear relationship indicative of logistic growth assumed by the majority of population models. A recent review of a wide range of taxa, spanning mammals, birds, fish, and insects showed that such nonlinear, decelerating density-dependent relationships are likely to be the norm [27]. Furthermore, these relationships are likely to have implications for population dynamics, potentially slowing population growth, driving large fluctuations in population size, and altering the population's response to perturbations. From an applied perspective, such nonlinear density-dependent relationships may have implications for parasite control; the response of the parasite population to the additional mortality imposed through a control programme may be affected by the functional form of such density-dependent regulating processes. 

The diversity of potential effects of density dependence on vectors of human disease makes it difficult to predict how the overall transmission of infection within a host population may be affected. Relating these experimental data to natural conditions is complicated as snail densities in the field are likely to be highly variable. Nevertheless the data do show that the effects of density dependence on infectious disease dynamics are not as straightforward as expected. The implications of such nonlinear density-dependent relationships for parasite control have yet to be examined using mathematical models.

## Figures and Tables

**Table 1:** Minimal adequate model for weight of snails containing all significant terms. Insignificant first-order terms were retained only if a second-order interaction containing that term was significant.

Fixed terms	Coefficient	*t*-value	*P* *-*value
Intercept	0.6	8.68	<.0001
Day	0.6	8.68	<.0001
Density	−0.0083	16.37	.0088
Exposure to parasite	−0.12	−3.33	.074
Day: density	−0.0003	−2.02	<.0001
Day: exposure	−0.0027	−3.00	.0033

Log likelihood = 88.34, DF = 117.

**Table 2:** Minimal adequate model for number of eggs laid per snail per week, determined using deletion testing as before.

Fixed terms	Coefficient	*t*-value	*P* *-*value
Intercept	16.76	8.84	<.0001
Day	−0.17	−4.27	<.0001
Density	−0.49	−6.21	<.001
Exposure to parasite	−2.86	3.23	.01
Day: density	0.005	2.96	.0038

Log likelihood = −349.99, DF = 104.
